# Discovery of IHMT-337 as a potent irreversible EZH2 inhibitor targeting CDK4 transcription for malignancies

**DOI:** 10.1038/s41392-022-01240-3

**Published:** 2023-01-16

**Authors:** Husheng Mei, Hong Wu, Jing Yang, Bin Zhou, Aoli Wang, Chen Hu, Shuang Qi, Zongru Jiang, Fengming Zou, Beilei Wang, Feiyang Liu, Yongfei Chen, Wenchao Wang, Jing Liu, Qingsong Liu

**Affiliations:** 1 Anhui Province Key Laboratory of Medical Physics and Technology, Institute of Health and Medical Technology, Hefei Institutes of Physical Science, Chinese Academy of Sciences, 230031 Hefei, Anhui P. R. China; 2 University of Science and Technology of China, 230026 Hefei, Anhui P. R. China; 3 Hefei Cancer Hospital, Chinese Academy of Sciences, 230031 Hefei, Anhui P. R. China; 4 Precision Medicine Research Laboratory of Anhui Province, 230088 Hefei, Anhui P. R. China

**Keywords:** Target validation, Drug development

## Abstract

Enhancer of zeste homolog 2 (EZH2), an enzymatic subunit of PRC2 complex, plays an important role in tumor development and progression through its catalytic and noncatalytic activities. Overexpression or gain-of-function mutations of EZH2 have been significantly associated with tumor cell proliferation of triple-negative breast cancer (TNBC) and diffuse large B-cell lymphoma (DLBCL). As a result, it has gained interest as a potential therapeutic target. The currently available EZH2 inhibitors, such as EPZ6438 and GSK126, are of benefit for clinical using or reached clinical trials. However, certain cancers are resistant to these enzymatic inhibitors due to its noncatalytic or transcriptional activity through modulating nonhistone proteins. Thus, it may be more effective to synergistically degrade EZH2 in addition to enzymatic inhibition. Here, through a rational design and chemical screening, we discovered a new irreversible EZH2 inhibitor, IHMT-337, which covalently bounds to and degrades EZH2 via the E3 ligase CHIP-mediated ubiquitination pathway. Moreover, we revealed that IHMT-337 affects cell cycle progression in TNBC cells through targeting transcriptional regulating of CDK4, a novel PRC2 complex- and enzymatic activity-independent function of EZH2. More significantly, our compound inhibits both DLBCL and TNBC cell proliferation in different preclinical models in vitro and in vivo. Taken together, our findings demonstrate that in addition to enzymatic inhibition, destroying of EZH2 by IHMT-337 could be a promising therapeutic strategy for TNBC and other malignancies that are independent of EZH2 enzymatic activity.

## Introduction

PRC2 (Polycomb repressive complex 2) was first discovered in Drosophila which epigenetically silences genes through chromatin remodeling.^1^ The core subunits of PRC2 include embryonic ectoderm development,^2^ suppressor of zeste 12 (SUZ12), RbAp46/48, and the catalytic subunit Enhancer of zeste homolog 2 (EZH2).^3^ The canonical role of EZH2 depends on amethyl donor, S-adenosyl-l-methionine (SAM), to exert methyltransferase activity, including the di- and trimethylation of lysine 27 on histone H3 (H3K27).^3^ Trimethylation of the lysine 27 residue on histone H3 tail (H3K27me3) is an epigenetic repression marker associated with cell differentiation and proliferation. EZH2 gain-of-function mutations and overexpression lead to aberrant H3K27me3 levels and result in tumorigenesis and metastasis.^4–7^ The gain-of-function point mutations in EZH2 have been found in over 20% diffuse large B-cell lymphoma (DLBCL) and 27% of follicular lymphoma (FL) patients.^8,9^ These mutations were considered as early clonal events in lymphomagenesis and are capable of sustaining disease progression. Mutations in EZH2 and BCL-6 collaborate together to promote the formation of GC-derived lymphomas.^6,8,10^ These findings indicated that EZH2 mutations collaborate together with other abnormalities to enforce a malignant germinal center phenotype. In addition to mutations, overexpression of EZH2 has also been observed in various solid tumors, including bladder cancer,^11,12^ gastric cancer,^13^ prostate cancer,^14^ melanoma,^15,16^ and breast cancer.^17,18^ The aberrant expression of EZH2 has been found associated with the disease progress in breast cancer, and leads to higher risk of invasion and metastasis in both prostate and breast cancers.^14,17,18^

Beyond its canonical PRC2-dependent role, EZH2 also acts in a PRC2 complex or methyltransferase activity-independent manner in cancers. Recently, many studies uncovered the noncanonical function of EZH2 as a transcription factor or co-activator, thus functions differently in these cancers.^19,20^ For example, EZH2 interacts with DNA-binding factors and transcriptional co-activators, alerts different genes expression in many cancers. In prostate cancer, EZH2 activates the transcription of androgen receptor (AR) gene during prostate tumorigenesis by directly occupying its promoter.^21^ In ER-positive breast cancer, EZH2 forms a transcriptional complex with ERa and beta-catenin to modulate different gene expression.^22^ In ER-negative basal-like breast cancer, EZH2 transcriptionally activates NF-kB independently of the RPC2 complex.^17^ Moreover, in triple-negative breast cancer (TNBC), EZH2 transcriptionally activates Notch1 to increase the number of tumor-initiating cells^23^ and alter Pax7 transcription through p38a-mediated phosphorylation of EZH2 at threonine 372 residue.^24^ Interestingly, EZH2 may itself activate RelB transcriptionally in the TNBC, adding to the complication. Treatment with estradiol, epoch and catenin, EZH2 combines to form a complex on MYC promoter that activates transcription independently of histone methyltransferase activity in TNBC.^17,22,25,26^ These findings suggest that EZH2 plays important roles in the transcription regulation in cancer cells and could be a promising therapeutic target for cancer therapy, including TNBC.

Most of the currently developed EZH2 inhibitors focus on targeting the enzymatic activity of EZH2 and exhibited antitumor activities, including Tazmestat (EPZ6438),^27^ CPI1205,^28^ GSK126,^7^ UNC1999,^29^ and EPZ005687.^30^ EPZ6438 has been granted approval from the US FDA for the treatment of patients with metastatic or locally advanced epithelioid sarcoma or relapsed/refractory FL. However, preclinical studies of these inhibitors shown limited effect on cancers that are independent of its enzymatic activity, including breast and prostate cancer, bearing high levels of EZH2.^21,22^ Moreover, the drug resistance emerged after long-term treatment with EZH2 inhibitors, and many of these resistances were caused by acquired EZH2 mutations that prevent drug binding or dependence on its transcriptional activity.^31,32^ Recently, proteolytic targeting chimeras (PROTACs) and hydrophobic tagging technology demonstrated the possibility of degrading EZH2 with promising results in preclinical studies of breast cancer.^33,34^ Based on this compelling evidence, we propose that in cancers that are more dependent on the noncanonical role of EZH2, it may be more effective to synergistically degrade EZH2 in addition to enzymatic inhibition. Thus, discovery and development of the new generation of inhibitors targeting EZH2 degradation may provide potential therapeutic benefit.

Recent studies have shown that overexpression of cyclin-dependent kinase CDK4 associated with Cyclin-D in a significant fraction of human breast cancers.^35,36^ CDK4 associates with D type cyclins and forms cyclin-D-CDK complexes to play a crucial role in different cancers, including breast cancer.^37^ The cyclin-D-CDK4/6 complexes perform sequential phosphorylation of the retinoblastoma protein (RB) and pRB-related proteins, thus regulate G1 phase progression in cell cycle.^38^ Highly specific CDK4/6 inhibitors have shown therapeutic potential particularly for ER or HER2-positive breast cancer,^2,39–41^ but not in TNBC, probably due to different mechanisms, including biomarkers expression or the bypassing of CDK4/6 activity through its noncatalytic role.^42–45^ Recently, the PROTACs of CDK4/6 shown promising effect in TNBC.^46^ These studies provide rationale for identifying the clinical biomarkers for CDK4/6 inhibitors and other effective therapeutic approach for TNBC treatment.

Here, through rationale design, chemical screening, and genetic studying, we discovered a novel EZH2 covalent inhibitor, IHMT-337, which selectively blocks the downstream function of EZH2 and degrades EZH2 protein through CHIP E3 ligase-mediated proteasome pathway. Furthermore, using a CUT&TAG assay in combination with pharmacological inhibition and genetic depletion, we found a novel nonenzymatic dependent function of EZH2 in the transcriptional regulation of CDK4, the cyclin-dependent kinase which play crucial role in breast cancer cell cycle progression, to impair TNBC cell proliferation. Moreover, in addition to inhibits DLBCL cell growth in vivo through its enzymatic inhibition, EZH2 degradation with IHMT-337 inhibits the proliferation of TNBC primary patient cells and the formation of primary organoids. Taken together, our findings provide a novel therapeutic strategy for TNBC and other EZH2 transcriptional activity dependent cancers.

## Results

### Identified IHMT-337 as a highly selective EZH2 inhibitor

**Fig. 1:**
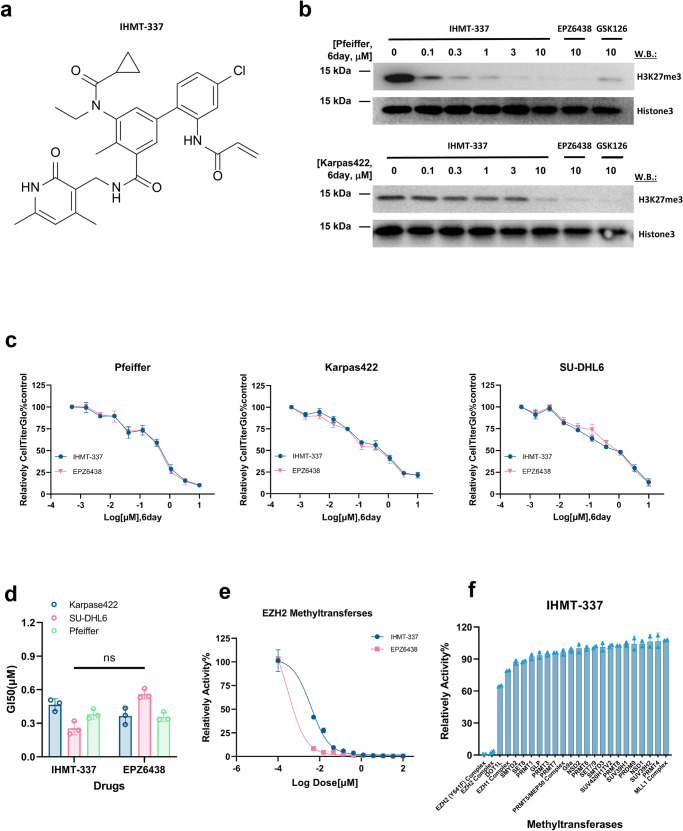
Characterization of IHMT-337 as a highly selective EZH2 inhibitor. **a** Chemical structure of IHMT-337. **b** EZH2 signaling studies: Target effects of IHMT-337 on EZH2 signaling in Pfeiffer and Karpas422 cell lines. EPZ6438 (the FDA-approved EZH2 inhibitor) was set as control. **c** Proliferation studies: Effects of 6-day IHMT-337 treatment of Pfeiffer,Karpas422 and SU-DHL6 cell lines. EPZ6438 was set as control. **d** The GI50 values (the concentrations that cause 50% growth inhibition) of IHMT-337 and EZP6438 to DLBCL cell lines were shown. **e** Biochemical assays: the effects of IHMT-337 on EZH2 methyltransferase activity on PRC2/EZH2 complex. **f** Methyltransferase selectivity profiling of IHMT-337 generated from the Hotpot approach. Data shown were representative of at least 2 independent experiments

In order to obtain a highly selective irreversible EZH2 inhibitor, we rationally designed and synthesized over 200 irreversible compounds, including IHMT-337 that targets the catalytic region of EZH2 and form covalent bond with the protein. Briefly, we identified some candidates using a EZH2-mediated E-cadherin promoter-driven firefly luciferase reporter screen system following the recent studies.^47,48^ Briefly, we cloned the 1.4 kb upstream regulatory region of E-cadherin promoter, which includes a EZH2 binding site, in order to generate a E-cadherin promoter-driven luciferase reporter. Introduction of this reporter into the HEK293T cells allowed us to confirm that EZH2 can lead to E-cadherin downregulation. As predicted, EZH2-mediated repression of the E-cadherin transcription was blocked by compounds including IHMT-337, which is consistent with the role of histone deacetylation during EZH2-mediated E-cadherin regulation. Then we evaluated our candidates for the ability to suppress the proliferation of EZH2-activating DLBCL cells, Pfeiffer (a EZH2-driving cell lines), in a 6-day assay, in which IHMT-337 were identified (Fig. 1a). We next investigated the effects of IHMT-337 on H3K27me3, the direct catalytic substrate of EZH2 by immunoblotting. Compared to EPZ6438 and GSK126, the two selective EZH2 inhibitors in clinical trials, IHMT-337 showed comparable effects on H3K27me3 levels in different DLBCL cell lines in a dose-dependent manner (Fig. 1b). The efficacies of IHMT-337 and EPZ6438 in other EZH2-dependent DLBCL cell lines were also investigated, and the results showed that both IHMT-337 and EZP6438 robustly impaired EZH2-dependent DLBCL cell proliferation with similar GI50s (Fig. 1c and d). In order to confirm the on-target effect of IHMT-337, we next displayed biochemical assays against purified EZH2 methyltransferase. As expected, both IHMT-337 and EPZ6438 showed similar effect on EZH2 enzymatic activity (Fig. 1e). To assess the selectivity of IHMT-337, we employed RBC’s Hotpot method to examine the effects of IHMT-337 on the enzymatic activity of 21 methyltransferases. The results revealed that IHMT-337 showed high selectivity to EZH2. In comparison to EPZ6438 with inhibition activity against both EZH2 and EZH1, IHMT-337 demonstrated excellent selectivity for EZH2 over a wide range of methyltransferases (Fig. 1f). Consistently, when we examined the effect of IHMT-337 on the trimethylation of other histone sites at the cellular level, the results showed that IHMT-337 specifically inhibited the methylation at H3K27 (Supplementary Fig. S1a). Taken together, these results suggest that IHMT-337 is a highly selective EZH2 inhibitor.

### IHMT-337 covalently binds to EZH2 at Cys663 residue

**Fig. 2:**
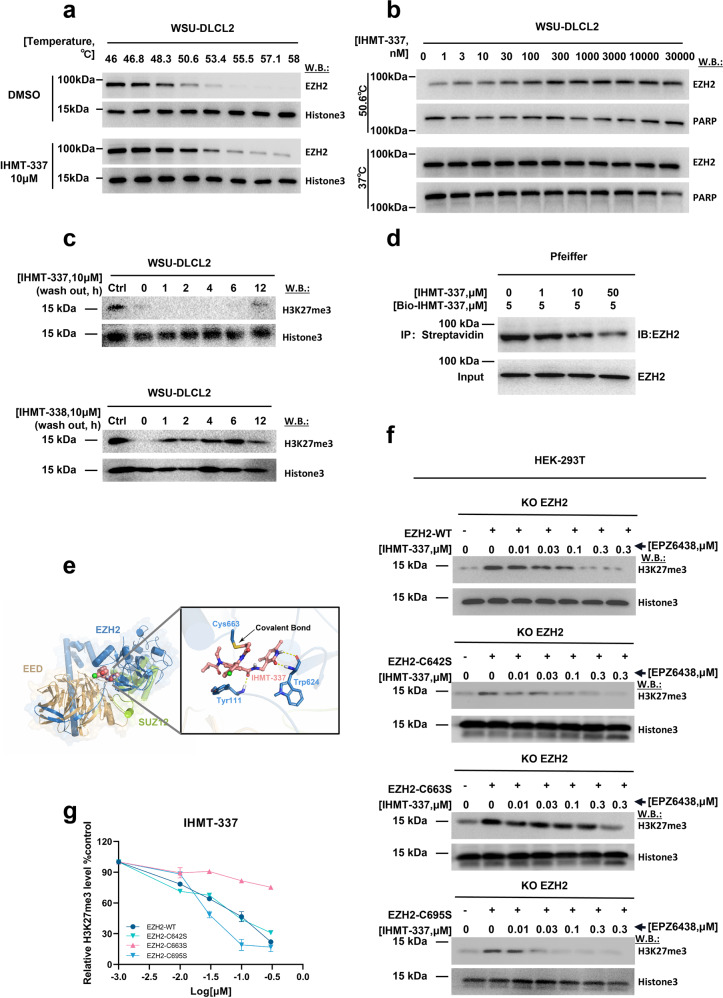
IHMT-337 covalently binds to EZH2 at Cys663 residue in SET domain. **a** The CETSA assay: The effect of IHMT-337 on the stability of the EZH2 protein in a temperature-dependent manner was investigated using WSU-DLCL2 cell lysate. **b** The CETSA assay: The effect of IHMT-337 on the stability of the EZH2 protein in a dose-dependent manner was investigated using WSU-DLCL2 cell lysate. **c** Washout assay: The effect of washout assay on signal pathway inhibition post-drug washout at different time points after using IHMT-337 and IHMT-338 treatment 72 h on WSU-DLCL2 cell line. **d** Target-engagement assay: Using Biotin-IHMT-337 and IHMT-337 to investigate the binding of IHMT-337 to EZH2 in Pfeiffer cells. **e** Predicted mode of binding of IHMT-337 to EZH2 based upon molecular modeling (PDB ID 5IJ7, chain B). **f** Using the HEK293T EZH2-KO cell line and plasmids with different mutations, investigation of the contribution of three cysteines in the SET domain to the direct binding of EZH2 and IHMT-337, the wt EZH2 was set as control. **g** The level of H3K27me3 was quantified and graphed. Shown are the representative results of three independent experiments

We next determined to know if IHMT-337 covalently binds to the SET region of the enzymatically active domain of EZH2 according to our design. As expected, the Cell Thermal Shift Test (CETSA) assays in temperature- and dose-dependent manner confirmed that IHMT-337 robustly improved EZH2 thermal stability, which demonstrated that IHMT-337 has the capacity to bind to EZH2 (Fig. 2a and b).

Then, to verify IHMT-337’s irreversibility, we developed and synthesized IHMT-338, an approximately isosteric analog (Supplementary Fig. S2a). While the negative control compound, IHMT-338, shown little to no inhibitory activity against EZH2 and the direct catalytic substrate of EZH2 (Supplementary Fig. S2b and S2c). We further tested the effect of washout assay, results showed no reduction in signal pathway inhibition for 12 h post-drug washout, indicating that IHMT-337 binds to EZH2 covalently (Fig. 2c). We performed a “target-engagement” assay to see if IHMT-337 can label EZH2. To this goal, we designed and synthesized Biotin-IHMT-337, a biotinylated homolog of IHMT-337, with a similar effect against EZH2 in both Pfeiffer and Karpas422 cells, compared to IHMT-337 and EPZ6438 (Supplementary Fig. S2d–2e). To confirm the target engagement of EZH2, we performed a competition assay in cell lysate treated with Biotin-IHMT-337 post 4 h treatment of IHMT-337 in dose-dependent manner. This investigation reveals that 50 μM IHMT-337 is adequate to label ~50% of EZH2 after 4 h (Fig. 2d), supporting the notion that EZH2 is a target of IHMT-337. The biochemical experiments with IHMT-337 and EZH2 and cellular target-engagement studies provide strong evidence that IHMT-337 covalently labels EZH2 in the cellular environment.

We next investigated the covalent binding site of IHMT-337 for EZH2. We initially generated vectors containing different EZH2 truncated genes, then performed CESTA tests in an overexpressed system. The results shown that IHMT-337 significantly increased the thermal stability of EZH2-C terminal but not EZH2-SET domain deletion or EZH2-N terminal, implying that there may be potential IHMT-337 binding sites on EZH2-SET domain (Supplementary Fig. S2f).

We next applied computer-aided structural analysis to confirm the binding mode of IHMT-337 in complex with EZH2 (PDB: 5IJ7,chain B) (Fig. 2e). The docking model shows that the scaffold of IHMT-337 is located at the ligand-binding pocket, the carbonyl and NH of pyridonemethyl-amide moiety form two stable hydrogen bonds with Trp624 residue of EZH2. In addition, carbonyl oxygen of the amide between pyridonemethyl-amide and methylaniline forms a pivotal hydrogen bond with Tyr111 residue. The cyclopropyl group, orientated toward the interface of EZH2 and EED, interacts with the side chains of Tyr111 and His199 residue. The methylaniline moiety and Phy665 also can form π–π stacking interaction. Most importantly, the warhead of acrylamide forms a covalent bond with Cys663 (Fig. 2e), which irreversibly inhibits the biological nature of EZH2.

Given the importance of cysteine residues in mediating the distinctive “Michael reaction” between the chemical compound and its direct target, we investigated the contribution of three cysteines in the SET domain to the direct binding of EZH2 and IHMT-337. By comparing the effect of IHMT-337 on the downstream of EZH2 in different mutants to determine the binding site. The EZH2-KO 293 T cell line was established to eliminate the background effect of endogenous EZH2 (Supplementary Fig. S2g). The H3K27me3 levels in HEK293T cells were mostly lost post knockout (KO) of EZH2 (Supplementary Fig. S4h). We then generated Cysteine site-mutations (C642S, C663S and C695S) in EZH2-SET region, and overexpressed them in HEK293T EZH2-KO cells, following treatment with a concentration gradient of IHMT-337, the direct binding site was determined by evaluation the inhibition of IHMT-337 on H3K27me3 recovery. The results showed that the C663 site mutant, but not the other two cysteine sites, blocked the restoration of H3K27me3 (Fig. 2f and g), suggesting that IHMT-337 covalently binds to EZH2 at Cys663 residue within the SET domain.

### IHMT-337 degrades EZH2 via CHIP-mediated ubiquitination pathway

**Fig. 3:**
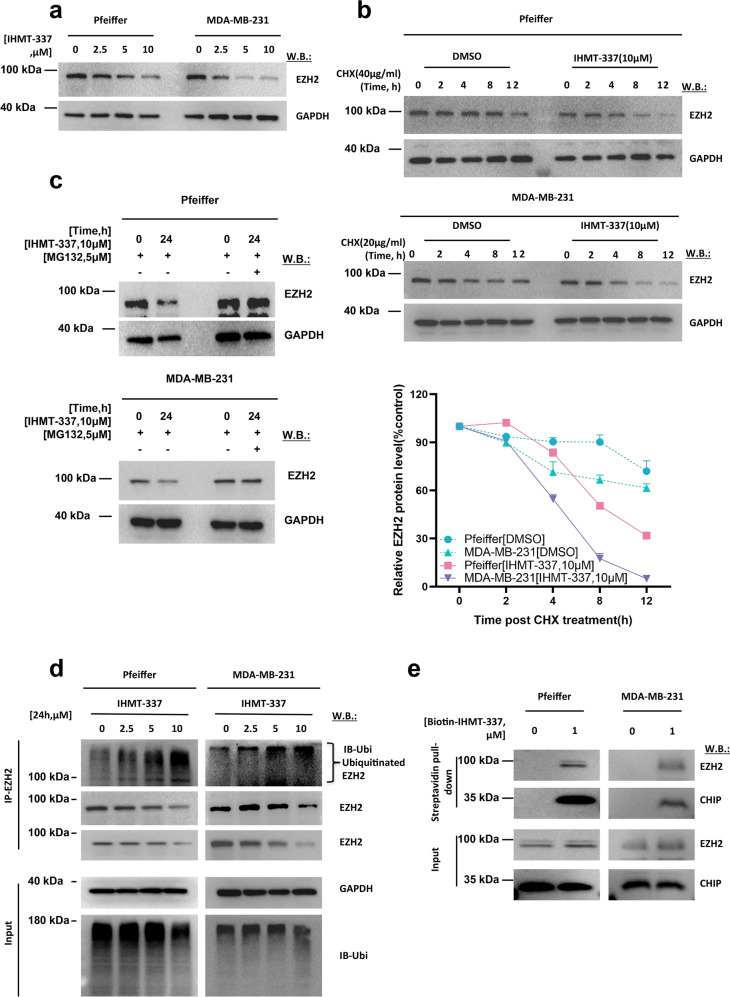
IHMT-337 degrades EZH2 via CHIP-mediated ubiquitination pathway. **a** Effects of 24 h IHMT-337 treatment on EZH2 protein levels in both Pfeiffer and MDA-MB-231 cells. **b** (Left panel) Pfeiffer and MDA-MB-231 cells were treated with CHX (40 μg/ml) with or without IHMT-337 treatment (10 μM) at the indicated time points. EZH2 and GAPDH protein levels were detected by western blotting. (Right panel) The half-life of EZH2 protein was quantified and graphed. Shown are the representative results of three independent experiments. **c** Pfeiffer and MDA-MB-231 cells were treated with IHMT-337 and with or without the proteasome inhibitor, MG132 (5 μM) at the indicated time points, EZH2 and GAPDH protein levels were detected by western blotting. **d** Pfeiffer and MDA-MB-231 cells were treated with IHMT-337 for 24 h at 0, 2.5, 5, and 10 μM. IP was performed with antibodies against EZH2, ubiquitin, EZH2, and GAPDH protein levels were detected by western blotting. **e** Cell lysates from Pfeiffer or MDA-MB-231 cells were treated with Biotin-IHMT-337 for 4 h at 0, 1 μM. IP was performed with Streptavidin bead through streptavidin-biotin interaction, and immunoblotting was performed with antibodies against EZH2 and CHIP

Previous studies have shown that covalent bonding between small molecule chemicals and proteins affect protein stability. Therefore, we examined the effect of IHMT-337 on the stability of EZH2 protein. As expected, we observed that IHMT-337 induces EZH2 degradation in both Pfeiffer and MDA-MB-231 cells (Fig. 3a). To exclude the possibility of transcription perturbation, we further checked EZH2 protein level in the presence of cycloheximide (CHX), a commonly used protein synthesis inhibitor in eukaryotic organisms. IHMT-337 was demonstrated to further shorten half-life of EZH2 when cells were treated with CHX than that of IHMT-337 treatment alone in different cell lines (Fig. 3b, Supplementary Fig. S3a), and We have performed QPCR to evaluate the mRNA changes of EZH2 post-IHMT-337 treatment in MDA-MB-231 cells, the results showed little to no effect of IHMT-337 on EZH2 at the transcriptional level (data not shown), suggesting that the loss of EZH2 induced by IHMT-337 at the posttranscriptional level has no effect on gene transcription. To explore the mechanism of EZH2 degradation by IHMT-337, we applied proteasome inhibitor MG132 in cells and found that EZH2 degradation induced by IHMT-337 was partially rescued (Fig. 3c). Furthermore, we observed increased poly-ubiquitination level of EZH2 following IHMT-337 treatment in both Pfeiffer and MDA-MB-231 cell lines (Fig. 3d). These results indicate that EZH2 is degraded through the ubiquitin-mediated proteasome pathway following IHMT-337 treatment.

Since a previously reported EZH2-degradable inhibitor GNA002 can degrade EZH2 through the CHIP E3 ligase-mediated ubiquitination degradation pathway.^49^ CHIP is one of the key players of the protein quality control system and mediates the poly-ubiquitination of misfolded or aggregated proteins for targeted degradation.^50,51^ We next investigated whether the CHIP E3 ubiquitin ligase played an essential role underlying the mechanism of IHMT-337-induced EZH2 ubiquitination. With transcriptome analyzing post-compound treatment, we found that CHIP is significantly increased at the transcriptome level (Supplementary Fig. S3b). We then confirmed the interaction between CHIP and EZH2 in different cell lines with streptavidin-biotin pull-down and observed increased binding post-IHMT-337 treatment (Fig. 3e, Supplementary Fig. S3c). Moreover, using co-immunoprecipitation (co-IP), we detected a robust association of EZH2 with CHIP in the presence or absence of IHMT-337, in a dose-dependent manner, which support the conclusion of the increased binding between EZH2 and CHIP post-IHMT-337 treatment in different DLBCL cell lines (Supplementary Fig. S3d). In addition, we have performed knockdown assay of CHIP in HEK293T cells, and the results further suggested that elimination of CHIP leads to partial rescue of EZH2 at the protein level, which is consistent with our previous finding that IHMT-337 recruits CHIP to mediate EZH2 degradation (Supplementary Fig. S3e). These results revealed that IHMT-337 as a novel agent that specifically and covalently bound to Cys663 residue within the EZH2-SET domain, leading to EZH2 protein misfolding or aggregation, then triggering EZH2 degradation through recruiting the Hsp70-interacting protein CHIP E3-mediated ubiquitination pathway in both DLBCL and TNBC.

### EZH2-mediated CDK4 transcriptional activity in TNBC

**Fig. 4:**
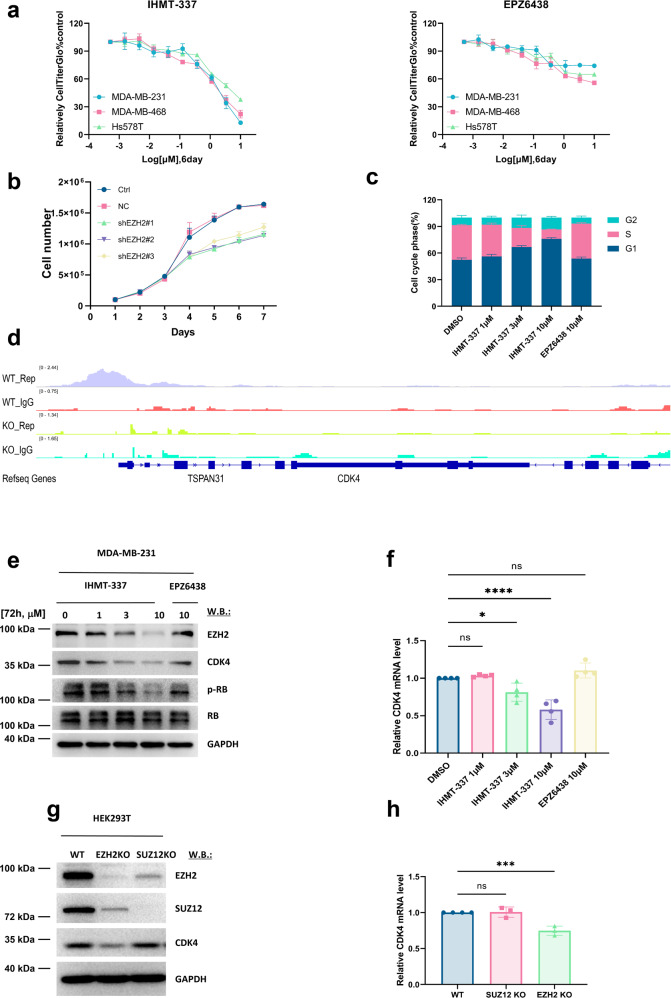
IHMT-337 inhibits breast cancer cell proliferation by degrading EZH2, a CDK4 transcription factor. **a** Proliferation studies: Effects of 6-day IHMT-337 treatment on proliferation of TNBC cell lines. EPZ6438 was set as control. **b** Proliferation studies: Effects of EZH2 knockdown on proliferation of MDA-MB-231 cells. **c** Cell cycle studies: Effects of IHMT-337 on cell cycle in MDA-MB-231 cell. EPZ6438 was set as control. **d** The CUT&TAG approach was used on HEK293T and HEK293T EZH2-KO cell lines to determine the sites of EZH2 binding to DNA. **e** Signaling studies: The inhibitory Effects of 72 h IHMT-337 treatment on cell cycle signaling in MDA-MB-231 cells. EPZ6438 was set as control. **f** Effects of 72 h IHMT-337 treatment of MDA-MB-231 cells on CDK4 transcriptional level. **g** Protein levels of EZH2 in HEK239T WT, HEK293T EZH2-KO, and HEK293T SUZ12 KO cells. **h** Transcriptional level of CDK4 in HEK239T WT, HEK293T EZH2-KO, and HEK293T SUZ12 KO cells

Recently, increasing number of studies uncovered the crucial role of nonenzymatic activity of EZH2 in different cancers progression. To identify the cancer types that could potentially benefit from covalent protein-degrading EZH2 inhibitor, IHMT-337, we first analyzed the TCGA database and results revealed that EZH2 is highly expressed in a variety of cancer types, especially breast cancer, which less dependent on PRC2 complex-related EZH2 methyltransferase activity. Among the four subtypes (basal-like, Her2, Luminal A, and Luminal B) of breast cancer we investigated, TNBC showed the highest level of EZH2 expression (Supplementary Fig. S4a and S4b). We then examined the effects of the irreversible IHMT-337 and the FDA-approved drug EPZ6438 on the proliferation of TNBC cell lines (Fig. 4a). The results demonstrated that IHMT-337 robustly suppressed TNBC cells growth, whereas EPZ6438 had little to no effect on TNBC cell proliferation. Colony formation experiment also confirmed that TNBC growth was impaired following IHMT-337 treatment (Supplementary Fig. S4c).

To investigate whether it was EZH2 degradation that caused by IHMT-337 influenced the proliferation of TNBC cells, we performed genetic knockdown of EZH2 and observed significant impairment of TNBC cell growth (Supplementary Fig. S4d and Fig. 4b). Colony formation experiments also confirmed that TNBC growth was suppressed following EZH2 genetic downregulation (Supplementary Fig. S4e). We then evaluated the effect of EZH2 degradation on TNBC cell cycle progression, we observed significant abnormalities on cell cycle-related pathways in TNBC cell line post-IHMT-337 treatment (Fig. 4c and Supplementary Fig. S4f).

As some studies have suggested the role of EZH2 in gene transcriptional regulation. We speculated that EZH2 can affect cell cycle progression by regulating the transcription of cell cycle-related proteins. To identify the target protein that responsible for cell cycle defect during the treatment of IHMT-337, we applied a CUT&Tag-based proteomic approach and found that EZH2 binds to the upstream region of CDK4 gene. Therefore, EZH2 associates with CDK4 promoter to regulate its transcription, suggesting that EZH2 may play as a transcriptional factor of CDK4 in TNBC (Fig. 4d).

We then investigated CDK4 and its downstream signaling, RB phosphorylation, following IHMT-337 treatment. Both immunoblotting and real-time PCR results indicate that EZH2 downregulation affects CDK4 transcription, resulting in decrease of CDK4 mRNA levels and subsequent protein levels (Fig. 4e and f). Biochemical assay of CDK4 excluded the effect of IHMT-337 on CDK4 enzymatic activity (Supplementary Fig. S5a), suggesting that IHMT-337 inhibits CDK4 levels through EZH2.Moreover, genetic knocking down of CDK4 showed impaired colony formation of TNBC, which is consistent with the pharmacological inhibition of EZH2 with IHMT-337 (Supplementary Fig. S5b–d). Recently, some studies reported that SUZ12 serves as a stabilizing factor (like a platform) for PRC2 via its VEFS domain.^52,53^ To confirm that EZH2 regulates CDK4 independent on its catalytic activity, we generated a PRC2-independent system through knocking out of SUZ12 in HEK293T cell, in which the PRC2 complex dissociated and EZH2 lost its main methyltransferase activity (Supplementary Fig. S5e and S5f). In this system, we found that CDK4 level remained unchanged post-KO of SUZ12, while both protein and mRNA levels diminished a lot following EZH2-KO (Fig. 4g and h), suggesting that EZH2 regulates CDK4 through its PRC2-independent function. In addition, CDK4 protein level was significantly upregulated by overexpression of EZH2 in TNBC (Supplementary Fig. S5g). Moreover, CDK4 protein level was restored with overexpression of WT EZH2 post-KO, similar effects were observed when using a catalytic domain (ΔSET2) deleted EZH2 (Supplementary Fig. S5h), these results suggest that EZH2-mediated transcriptional regulation of CDK4 is independent of PRC2 complex, thus revealing a novel transcriptional activity of EZH2.

Taken together, these results suggest that IHMT-337 blocks cell cycle progression of TNBC cells through inhibiting EZH2-mediated transcriptional regulation of CDK4, thus revealed a novel transcriptional activity of EZH2 in TNBC.

### IHMT-337 inhibits tumor cell growth in different preclinical models

**Fig. 5:**
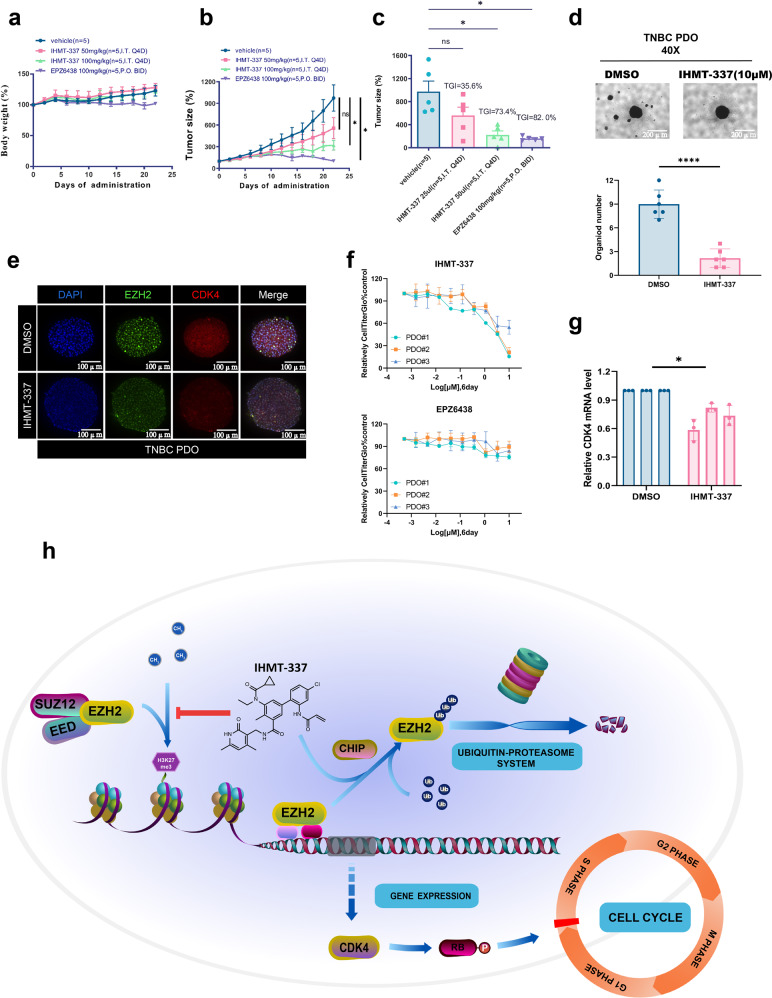
IHMT-337 inhibits cell proliferation in different preclinical models in vitro and in vivo. **a** Body weight change in mice for each twice-daily dosing group of IHMT-337 and EPZ6438. Initial body weight was set as 100%. Comparison of the final tumor weight in each group of 22-day treatment period. **b** Relative tumor size measurements of Pfeiffer xenograft mice after IHMT-337 and EPZ6438 treatment. **c** Effects of 22 days IHMT-337 treatment on growth of Pfeiffer xenograft tumor model were determined. EPZ6438 was set as control. **d** Effects of 72 h IHMT-337 treatment on TNBC PDO models. **e** The inhibitory effects of IHMT-337 on protein levels of EZH2 and CDK4 in TNBC PDOs were determined by confocal assays. **f** The inhibitory effects of IHMT-337 on proliferation of TNBC PDOs were determined. EPZ6438 was set as control. **g** Transcriptional level of CDK4 in TNBC PDOs with or without IHMT-337 treatment were determined by Q-PCR. **h** IHMT-337 affects cell cycle progression through targeting transcriptional regulating of CDK4

To assess the in vivo antitumor activity of IHMT-337, we treated the xenograft mouse model inoculated with Pfeiffer cells with different dosages of IHMT-337 and intratumoral injection for 22 days. IHMT-337 displayed dose-dependent tumor growth suppression with a tumor growth inhibition (TGI) rate of 73.4% and no apparent body weight loss was observed (100 mg/kg intratumoral injection, Q4D), displayed similar potency to EPZ6438 (Fig. 5a–c).

To further explore the therapeutic benefits of IHMT-337 in more solid tumors that are independent of EZH2 catalytic activity, we then investigated the effects of our compound in primary TNBC patient-derived organoid (PDOs) in vitro. We obtained primary TNBC patient samples and established the PDOs models to mimic the biological characteristics of the primary tumors. Most of the original pathological characteristics were maintained according to the published protocols.^54^ We observed larger sizes and a smaller number of organoids formation following IHMT-337 treatment (Fig. 5d and e), suggesting that IHMT-337 may inhibit TNBC primary cell growth. We then observed robust growth inhibition in IHMT-337 treated PDOs versus EPZ6438 (Fig. 5f). Moreover, the immunofluorescence and confocal assays shown the diminished signal of CDK4 following the loss of EZH2 post-IHMT-337 treatment (Fig. 5e). The real-time PCR assay confirmed these results (Fig. 5g). These results are consistent with the finding that IHMT-337 blocks CDK4 transcription following EZH2 degradation in TNBC cells (Fig. 4e and f).

In summary, a novel irreversible EZH2 inhibitor, IHMT-337, was rationally designed and synthesized. In vitro analysis showed that this compound is able to form covalent interaction with EZH2 and induce EZH2 protein degradation through CHIP-mediated ubiquitination pathway. Further functional study revealed a novel role of EZH2 in the transcription regulation of CDK4 with its noncatalytic activity (Fig. 5h). We confirmed the antitumor efficacy in both DLBCL xenograft and TNBC PDO preclinical models. This study highlighted the therapeutic potential of inhibiting both catalytic and noncatalytic functions of EZH2 by protein degradation strategy for the treatment of TNBC and other malignancies.

## Discussion

Overexpression or gain-of-function mutation of EZH2 with high levels of trimethylation on H3K27 have been found in both hematologic malignancies and solid tumors, which are correlated with poor prognosis and drug resistance. Recently, an increasing number of studies revealed that EZH2 promotes tumor development and progression through its methyltransferase activity-independent functions. In addition to the PRC2 complex-dependent activity, EZH2 can also serve as a transcriptional factor. For example, in chronic lymphocytic leukemia, EZH2 occupies the Insulin Like Growth Factor 1 Receptor (IGF1R) promoter region along with Myc, upregulates IGF1R expression, and activates the downstream PI3Ks.^55^ In colon cancer cells, PCNA-associated factor (PAF) and EZH2 were found to be involved in Wnt signaling extra activation.^56^ Moreover, TRIM28 (also known as KRAB-associated protein 1 or KAP1) has previously been shown to promote breast cancer proliferation and metastatic progression. EZH2 interacts with the SWI/SNF chromatin remodeling complex and TRIM28 subunits to form a complex distinct from PRC2 in MCF7 ER-positive breast cancer cells.^57^ Together, EZH2 and TRIM28 activate a set of stemness genes that promote mammosphere formation. These findings suggest that the transcriptional activity of EZH2 is important in the occurrence and progression of cancers, including breast cancer.

Inhibitors targeting EZH2 enzymatic activity provided benefit in clinical use, such as EPZ6438^27^ and GSK126,^7^ which have been approved or in clinical trials for sarcoma or lymphoma. However, some cancers are independent of its catalytic activity and do not response after treatment or easily acquired drug resistance with EZH2 inhibitors. Therefore, the methyltransferase-independent functions of EZH2 limits the efficacy of current EZH2 inhibitors. Studies have shown that EPZ6438, the FDA-approved EZH2 inhibitor, has marginal effect on the growth of TNBC cells.^58^ Recently, proteolytic targeting chimeras (PROTACs) and hydrophobic tagging technology demonstrated the possibility of degrading EZH2 with promising results in preclinical studies of breast cancer.^33,34^ Thus, an irreversible inhibitor with enzymatic inhibition combined with depletion or degradation of EZH2 has potential therapeutic benefit for EZH2-highly expressed TNBC or other related malignancies.

Here, combining rationale design, chemical screening and genetic studying, we discovered a novel EZH2 covalent inhibitor, IHMT-337, which selectively impairs the enzymatic activity of EZH2 and degrades EZH2 through CHIP E3 ligase-mediated proteasome pathway. As we excepted, compared to its reversible analog, IHMT-337 exerts high potency in malignancies in which EZH2 functions as a non-methyltransferase, such as breast and prostate cancer. Furthermore, using a CUT&TAG assay, in addition to pharmacological inhibition and genetic depletion approaches, we found a new PRC2 complex- and EZH2 catalytic activity-independent function of EZH2, through transcriptional regulating CDK4 to disrupt the cell cycle progression of TNBC cells. CDK4 had been reported to be highly expressed and associates with D type cyclins and plays a crucial role in breast cancer.^37^ However, the basal-like TNBC was reported to be insensitive to CDK4/6 inhibitors due to complicated mechanisms, including regulating the cyclin E/CDK2 activity in a noncatalytic manner by CDK4. Therefore, the remained high cyclin E/CDK2 activity post-CDK4/6 inhibitors treatment causes bypassing of requirement of CDK4/6 activity,^42–45^ and the PROTACs of CDK4/6 shown promising effect in TNBC cells,^46^ thus presenting the need for identification of new effective therapeutic approach that correlated with CDK4 downregulation or degradation for TNBC treatment. Here, we revealed a novel transcriptional regulation function of EZH2 in TNBC, to our knowledge, this is the first time that CDK4 is reported as a substrate of EZH2, thus expanding the clinical utility of EZH2 degrader. We found that EZH2 is highly expressed and acts as a transcription factor in TNBC through regulating CDK4, degrades EZH2 with IHMT-337 leads to CDK2 decreasing simultaneously (data bot shown), which provides evidence for clinical utility of our compound for basal-like TNBC.

Other than CDK4 inhibition, we also found some reported or new tumor suppressor genes that may potentially regulated by EZH2 degradation induced by IHMT-337 in our transcriptomic data, suggesting IHMT-337 induced degradation of EZH2 results in complicated transcriptional regulation. Our future efforts will be focus on EZH2’s new function in regulation of those new factors. In addition, although there are some cross-talking between the knockout of EZH2 and SUZ12 (Fig. 4g, Supplementary Fig. S4h), which is consistent with some recent studies,^59,60^ we did not find significant changes in either SUZ12 or EED at the transcriptome level, suggesting the on-target effect of IHMT-337 on EZH2. We further tested EED and SUZ12 levels in a EZH2-KO cell line with or without IHMT-337 and found that both SUZ12 and EED were significantly reduced post-KO of EZH2. However, there is no further loss of SUZ12 post-IHMT-337 treatment till 3 µM (data not shown), which conformed that the effect we saw with SUZ12 or EED is a secondary effect from protein complex destabilization caused by covalent inhibitor IHMT-337.

Moreover, pharmacological inhibition and degradation of EZH2 with IHMT-337 leads to the death of both DLBCL cell in vivo and TNBC primary patient cells and aberrant formation or growth of primary PDOs in vitro. Taken together, therapeutic targeting EZH2 by promoting its degradation is an effective approach to overcome low drug efficacy and resistance due to its nonenzymatic activity. Thus, our study provides a potential therapeutic strategy for TNBC and other EZH2 transcriptional activity dependent malignancies.

As the mechanisms underlying TNBC progression are complicated, in addition to EZH2-targeting as a monotherapy, there are numerous combinatory therapeutic approaches that can be investigated. For example, we found that there are still some H3K27me3 left after KO of endogenous EZH2, which is consistent with some recent studies showed that EZH1 targets a subset of EZH2 genes,^61^ and the functional redundancy allows EZH1 to partially compensate for the loss of EZH2 during lineage commitment and EZH2-KO cells.^62,63^ Therefore, combination of an EZH2 inhibitor with EZH1 inhibitor or degrader, or combination with immune checkpoint inhibitors or antibodies, or combination with kinase inhibitors, such as PI3K inhibitors,^64^ would be new potential therapeutic directions.

## Materials and methods

### Cell lines

Human breast cancer cell lines (MDA-MB-231, MDA-MB-468, and Hs578T), DLBCL cell line (Pfeiffer, Karpas 422, WSU-DLCL2 and SU-DHL6), and HEK293T were purchased from Cobioer Biosciences Co., Ltd (Nanjing, China). Pfeiffer, Karpas 422, WSU-DLCL2 and SU-DHL6 cell lines were cultured in RPMI-1640 medium (Corning, Midland, NY, USA) with 10% FBS (VivaCell, Shanghai, China), and MDA-MB-231, MDA-MB-468 and HEK293T were cultured in DMEM medium (Corning) with 10% FBS (VivaCell, Shanghai, China), Hs578T were cultured in MEM medium (Corning, Midland, NY, USA) with 10% FBS(VivaCell, Shanghai, China) and supplemented with 1% penicillin/streptomycin (v/v).

### Chemical reagents

IHMT-337, Biotin-337 and IHMT-338 were synthesized in the lab; EPZ6438 (CAS #231277-92-2), MG132 (CAS#133407-82-6) and Cycloheximide (CAS#66-81-9) were purchased from Medchemexpress.

### Antibodies

The EZH2 (D2C9) Rabbit mAb (#5246), SUZ12 (D39F6) XP® Rabbit mAb (#3737), PARP (46D11) Rabbit mAb (#9532), Ubiquitin (P4D1) Mouse mAb (#3936), CDK4 (D9G3E) Rabbit mAb (#12790), Phospho-Rb (Ser807/811) (D20B12) Rabbit mAb (#8516), Tri-Methyl-Histone H3 (Lys27) (C36B11) Rabbit mAb (#9733), Tri-Methyl-Histone H3 (Lys4) (C42D8) Rabbit mAb (#9751), Tri-Methyl-Histone H3 (Lys9) (D4W1U) Rabbit mAb (#13969) and Tri-Methyl-Histone H3 (Lys79) (E8B3M) Rabbit mAb (#74073) were obtained from Cell Signaling Technology. Anti-EZH2 antibody (ab283270) and anti-STUB1/CHIP antibody (EPR4447) were obtained from Abcam. Anti-Flag (F2555-100UL) were obtained from Sigma (Danvers, MA, USA). GAPDH(60004-1-Ig), RB(10048-2-Ig) and Histone H3(17168-1-AP) were obtained from Proteintech. All antibodies were diluted according to the instructions and used for western blotting experiments.

### Biochemical assay

The enzymatic inhibition assays of IHMT-337 were carried out using the Hotpot technology following the manufacturer instructions (RBC).

### Primary cells and organoids culture

Human primary TNBC patient samples were obtained under approval of the Chinese Academy of Sciences Institutional Review Board. Human cell lines were authenticated within 6 months of manuscript preparation through cell line short tandem repeat (STR) profiling (Molecular Diagnostics Laboratory, Chinese Academy of Sciences). The organoid culture protocol and organoid culture media produced by STEMCELL Technologies are used for the culture of patient-derived organoids (PDOs) models.

### Covalent docking study

CovalentDock,^65^ written based on Autodock, was implemented to predict the interaction of IHMT-337 binding to EZH2. The crystal structure of human EZH2 used for this docking job was obtained from the Protein Data Bank (PDB ID 5IJ7, chain B) and was prepared by using PyMOL, including removing water molecules and adding hydrogens. Then minimization was performed to avoid local collision. A grid box of 60 × 60 × 60 points centering on the coordinate of 13.82, 3.68, and 281.45 was implemented, which encloses the whole binding pocket. Other parameters were set as default during the docking.

### Real-time quantitative PCR

Total RNA from organoids were isolated with the RNeasy kit (TIANGEN, DP430). A total of 0.5–1 μg RNA was used to synthesize complementary DNA using Script Reverse Transcription Supermix (TAKARA, RR037). Quantitative PCR was performed with Power SYBR Green PCR Master Mix (TAKARA, RR820A). The primer sequences used are listed in Supplementary Table 1.

### Plasmid construction

PCR reactions were performed using the PrimeSTAR Max DNA Polymerase (TAKARA). All primer sequences used are listed in Supplementary Table 1. EZH2-Flag plasmid was purchased from Sino Biological. To generate pcDNA4.1-EZH2-C, pcDNA4.1-EZH2-N and pcDNA4.1-EZH2-ΔSET, the fragments of truncated EZH2 genes were amplified by PCR and insert into pcDNA4.1 vector (Thermo Fisher) at BamHI and EcoRI sites using T4 DNA ligase (TAKARA).

To generate Flag-EZH2-C642S, Flag-EZH2-C663S, and Flag-EZH2-C695S, site-directed mutagenesis was performed using the Mut Express II Fast Mutagenesis Kit V2 (Vazyme Biotech). All constructs were checked by Sanger sequencing.

### Cut&Tag assay

CUT&Tag was performed as previously described^66^ with Hyperactive In-Situ ChIP Library Prep Kit for Illumina kit (Vazyme Biotech, TD901). Briefly, cells were treated with 10 μl pre-washed ConA beads for 10 min before adding 0.5 μg antibody and incubated at room temperature for 2 h. After washing with dig-wash buffer, samples were incubated for 30 min at room temperature with 0.5 μg secondary antibody. After two more washes, added 0.58 μl pG–Tn5 and incubated at RT for 1 h, washed twice more, added 300 μl tag mentation buffer, and incubated at 37 °C for 1 h. Terminated the reactions, extracted the samples with phenol-chloroform and ethanol, amplified the libraries with PCR, and sequenced the libraries according to the manufacturer’s instructions.

### Transcriptome sequencing and analysis

RNA from the pfeiffer was extracted using TRIzol (Takara). RNA integrity was assessed using the Bioanalyzer 2100 system (Agilent Technologies), and high-quality samples were chosen for library preparation. After cluster generation, the library preparations were sequenced on an Illumina Novaseq platform (NOVOGENE Company Limited, China) to obtain 150 bp paired-end reads. Hisat2 was used to align the clean paired-end sequences to the reference human genome. The DESeq2 R package (1.30.1) was used with standard settings to conduct differential expression analysis. Genes were classified as differentially expressed provided the FDR adjusted P-value(Benjamini and Hochberg’s approach) <0.1.Corrected *P*-value of 0.1 and absolute foldchange of 2 was set as the threshold for significantly differential expression.The cluster profile R program chose differentially expressed genes (corrected *P*-value of 0.1) for GO and KEGG enrichment analysis, GO terms and KEGG pathways with *P*-values less than 0.01 were defined as significantly enriched.

### CETSA assay

Cells were harvested and resuspended in culture medium at a cell density of 5 × 10^6^ cells per ml before being seeded into T25 flask (Coring Plastics) for the CETSA assay. IHMT-337 or vehicle (DMSO) was added to cell lysates and incubated for 1 h. Samples were then divided into 100 μl aliquots in 0.2 ml PCR tubes and heated in a PCR machine (ProFlex, Applied Biosystems) for 3 min at indicated temperatures, followed by 3 min of cooling at RT. Samples were then freeze-thawed for three cycles and centrifuged to remove the precipitates before analyzing the remaining soluble fraction with Western blot.

### Colony formation assay

Cells were seeded in six-well plates for 24 h before being treated with IHMT-337 at the indicated concentrations. The colonies were stained with crystal violet after 14 days.

### Gene expression knockdown and gene knockout

EZH2 and CDK4 knockdown lentivirus were purchased from GenePharma. Knockout of EZH2 and SUZ12 was performed with sgRNAs In Vitro one-step Transcription Kit (Novoprotein, E369-01A). Primer sequences are listed in Supplementary Table 1. Incubated 0.2 μg sgRNA and 5 μl Cas9 protein (Novoprotein, E379-01) in Cas9 buffer for 2 h at 37 °C to generate Cas9 RNP complex. Mixed 3 μl lipofectamine 2000 (GenePharma,11668-027) transfection reagent, 25 μl Opti-MEM reduced serum medium and Cas9 RNP complex well, then incubated for 15 min at room temperature. The transfection complex was gently dropped into cells with fresh culture media and cultivated for 48–72 h.

### Cell cycle analysis and flow cytometry (FACS)

MDA-MB-231 were seeded in 6-well plates at a density of 1 × 10^5^ cells/well overnight. On the following day, cells were treated with vehicle (DMSO) or IHMT-337 for 72 h. Cells were incubated with 0.5 ml of PI/RNase staining buffer for 15 min at room temperature (RT). The DNA content and cell cycle distribution were assessed by FACScan laser flow cytometry (BD). The data were analyzed using Flowjo and Prism software.

### Immunofluorescence or confocal microscopy

Human primary TNBC patient-derived organoids (PDOs) were incubation on a microscope slide pre-coated with poly-L lysine for 1 h. Adhered cells were then fixed by 4% formaldehyde for 0.5 h followed by blocking with 1% BSA and permeabilized with 0.5% Triton-X 100 and incubated with indicated primary antibodies for 1 h at RT. Cells were washed three times with 1× PBS and incubated for 60 min with donkey anti-mouse-Dylight 488 and goat anti-Rabbit-Dylight 594. Cell nuclei were stained by DAPI dye (1:1000). And slides were imaged with a laser confocal microscope (Olympus). Representative immunofluorescence or confocal microscopy images (×40 or ×60) were visualized for endogenous EZH2 (green) and CDK4 (red), DAPI was used to visualize cell nuclei (blue).

### Pfeiffer xenograft tumor model

Five-week-old female nu/nu mice were purchased from the Nanjing Biomedical Research Institute of Nanjing University (Nanjing, China). All animals were housed in a specific pathogen-free facility and used according to the animal care regulations of Hefei Institutes of Physical Science, Chinese Academy of Sciences (Hefei, China). In vivo studies were under the approvement of the Hefei Institutes of Physical Science ethics committee, Chinese Academy of Sciences. Five million Pfeiffer cells in PBS were formulated as a 1:1 mixture with Matrigel (BD Biosciences) and injected into the subcutaneous space on the right flank of nu/nu mice. Animals were then randomized into treatment groups of five mice each for efficacy studies. Intratumoral injection administration was initiated when tumors reached a size of 200–400 mm^3^. IHMT-337 was delivered in a HKI suspension (0.5% methocellulose/0.4% Tween80 in ddH2O) by intratumoral injection (Q4D). A range of doses of IHMT-337 or its vehicle was administered as indicated in the Fig. 5 legend. Body weights and tumor growths were measured daily after IHMT-337 treatment. Tumor volumes were calculated as follows: tumor volume (mm^3^) = [(W2 × L)/2] in which width (W) is defined as the smaller of the two measurements and length (L) is defined as the larger of the two measurements.

### Statistical analysis

All results are presented as the means ± SEM and Student’s *t*-test was used for statistical analysis among the different groups; IC50 and GI50 values were calculated using Prism 9.0 (Graph Pad Software, San Diego, CA, USA) using the normalized dose response curve for inhibition (variable slope).

## Supplementary information

SupplementaryMaterials-SIGTRANS-06784R1

## Data Availability

The data that support the findings of this study are available from the lead corresponding author upon reasonable request.
