# Supplementary material for: Discovery of IHMT-337 as a potent irreversible EZH2 inhibitor targeting CDK4 transcription for malignancies
Source: Signal Transduct Target Ther. 2023 Jan 16;8:18. doi: 10.1038/s41392-022-01240-3 (PMC9841011; doi:10.1038/s41392-022-01240-3)
Supplement: Supplementary file 1 — SupplementaryMaterials-SIGTRANS-06784R1 [file 41392_2022_1240_MOESM1_ESM.pdf]

SUPPLEMENTARY MATERIALS

Discovery of IHMT-337 as a potent irreversible EZH2 inhibitor targeting CDK4 transcription for malignancies

Husheng Mei[1,2], Hong Wu[1,3], Jing Yang[1,3], Bin Zhou[1,2], Aoli Wang[1,3], Chen Hu[1,3], Shuang Qi[1,3], Zongru Jiang[1,3], Fengming Zou[1,3], Beilei Wang[1,3], Feiyang Liu[1,3], Yongfei Chen[1,3], Wenchao Wang[1,3,*], Jing Liu[1,3,**], Qingsong Liu[1,2,3,4,***]

[1] Anhui Province Key Laboratory of Medical Physics and Technology, Institute of Health and Medical Technology, Hefei Institutes of Physical Science, Chinese Academy of Sciences, Hefei, Anhui 230031, P. R. China

[2] University of Science and Technology of China, Hefei, Anhui 230026, P. R. China

[3] Hefei Cancer Hospital, Chinese Academy of Sciences, Hefei, Anhui 230031, P. R. China

[4] Precision Medicine Research Laboratory of Anhui Province, Hefei, Anhui 230088, P. R. China

These authors contributed equally to this work：Husheng Mei, Hong Wu, Jing Yang, Bin Zhou.

***Corresponding authors. Institute of Health and Medical Technology, Hefei Institutes of Physical Science, Chinese Academy of Sciences, Hefei, Anhui 230031, P. R. China. E-mail address：qsliu97@hmfl.ac.cn(Q.Liu)

**Corresponding authors. Institute of Health and Medical Technology, Hefei Institutes of Physical Science, Chinese Academy of Sciences, Hefei, Anhui 230031, P. R. China. E-mail address：jingliu@hmfl.ac.cn(J.Liu)

*Corresponding authors. Institute of Health and Medical Technology, Hefei Institutes of Physical Science, Chinese Academy of Sciences, Hefei, Anhui 230031, P. R. China. E-mail address：wwcbox@hmfl.ac.cn(W.Wang)

Table of Contents

1. Suppl. Table S1. Primers---S3

2. Suppl. Fig. S1. Methyltransferase selectivity profiling of IHMT-337 generated from Pfeiffer cells--S4

3. Suppl. Fig. S2. IHMT-337 covalently binds to EZH2 at SET domain------------S5

4. Suppl. Fig. S3. Transcriptome analyzing identified CHIP as the E3 ligase that mediate EZH2 degradation post-IHMT-337 treatment ------------------------------S6

5. Suppl. Fig. S4. EZH2 is a CDK4 transcription factor -------------------------------S7

6. Suppl. Fig. S5. TNBC growth was suppressed following the pharmacological and genetic deletion of EZH2 or CDK4 --S8

Supplementary Table S1 Primers

primer name	sequence
EZH2 KO sgRNA-F	CaccgAGAACAGGTCTTCGGCTTCG
EZH2 KO sgRNA-R	AaacTCTTGTCCAGAAGCCGAAGC
SUZ12 KO sgRNA-F	CaccgACCGGTGAAGAAGCCGAAAA
SUZ12 KO sgRNA-R	AaacTTTTCGGCTTCTTCACCGGT
EZH2 N-F	CCGGGATCCATGGATTACAAGGATGACGACGATAAGGGT
EZH2 N-R	AGCGGTGAGAGCAGCAGCAAACGCTCGAGCGG
EZH2 C-F	CCGGGATCCATGGAGCGGATAAAGACCCCA
EZH2 C-R	TTAAGGGATTTCCATTTCTCTTTCGCGCTCGAGCGG
EZH2 SET△-F	CCGGGATCCATGGAGCGGATAAAGACCCCAC
EZH2 SET△-R	TTTGGAGCCCCGCTGAATACCGCTCGAGCGG
CDK4 F	ATGGCTACCTCTCGATATGAGC
CDK4 R	CATTGGGGACTCTCACACTCT
GAPDHRT1 R	GGAGCGAGATCCCTCCAAAAT
GAPDHRT1 F	GGCTGTTGTCATACTTCTCATGG

Supplementary Figure 1

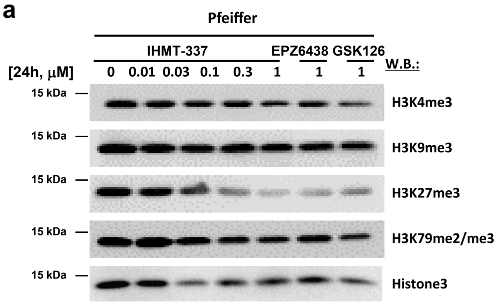

Supplementary Figure S1 a. Methyltransferase selectivity profiling of IHMT-337 generated from Pfeiffer cells. Data shown were representative of at least 2 independent experiments.

Supplementary Figure 2

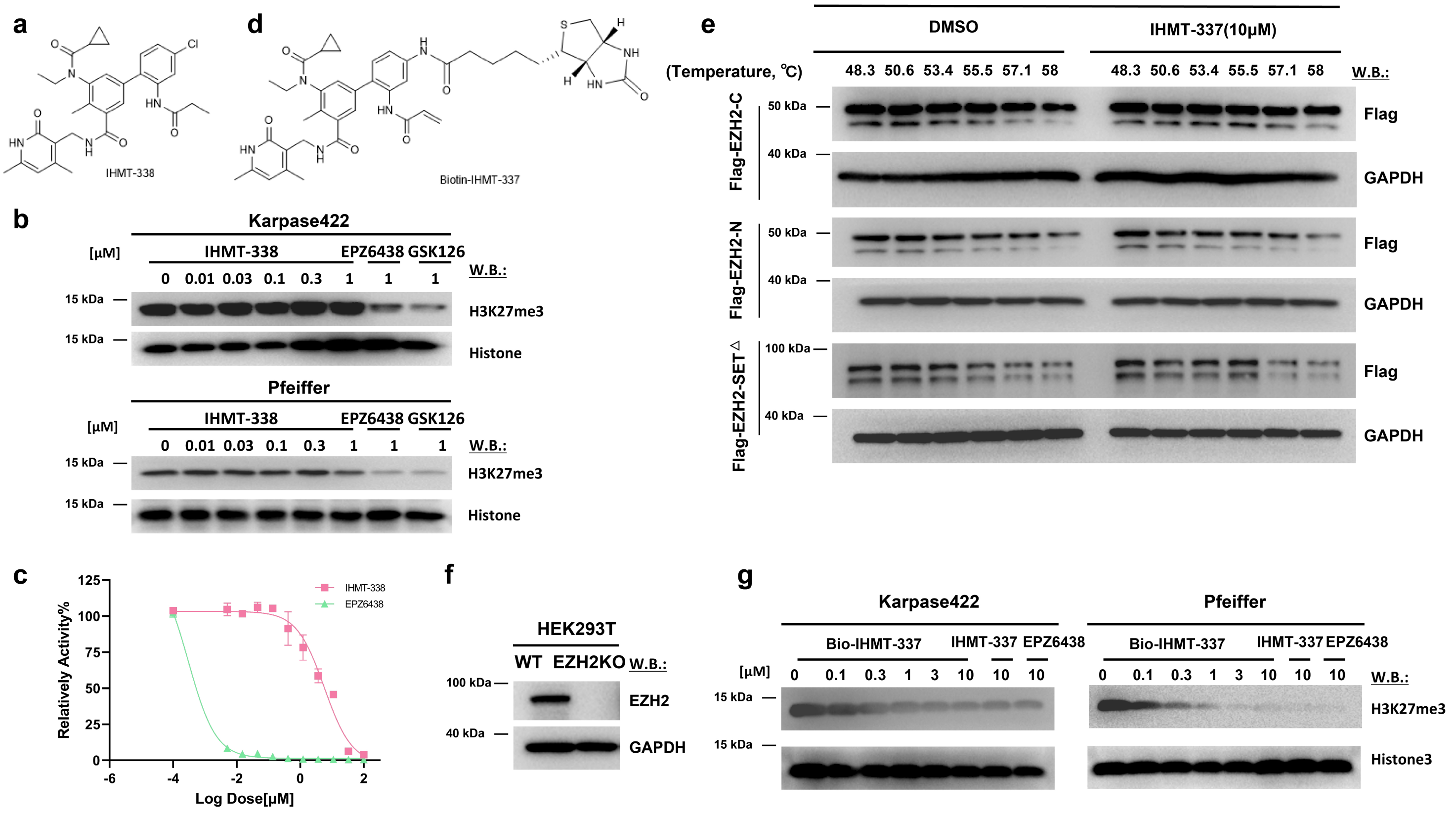

Supplementary Figure S2. IHMT-337 covalently binds to EZH2 at SET domain. **a.** Chemical structure of IHMT-338. **b.** Target effects of IHMT-338 on EZH2 signaling in DLBCL cells. EPZ6438 (the FDA approved EZH2 inhibitor) and GSK126 was set as control. **c.** Biochemical assays of IHMT-338 against purified EZH2 methyltransferase. **d.** Chemical structure of Biotin-IHMT-337. **e.** CETSA experiments on different fragments of EZH2. **f.** Protein levels of EZH2 in HEK293T EZH2 KO cells. **g.** Target effects of Biotin-IHMT-337 on EZH2 signaling in DLBCL cells. IHMT-337 and EPZ6438 was set as control.

Supplementary Figure 3

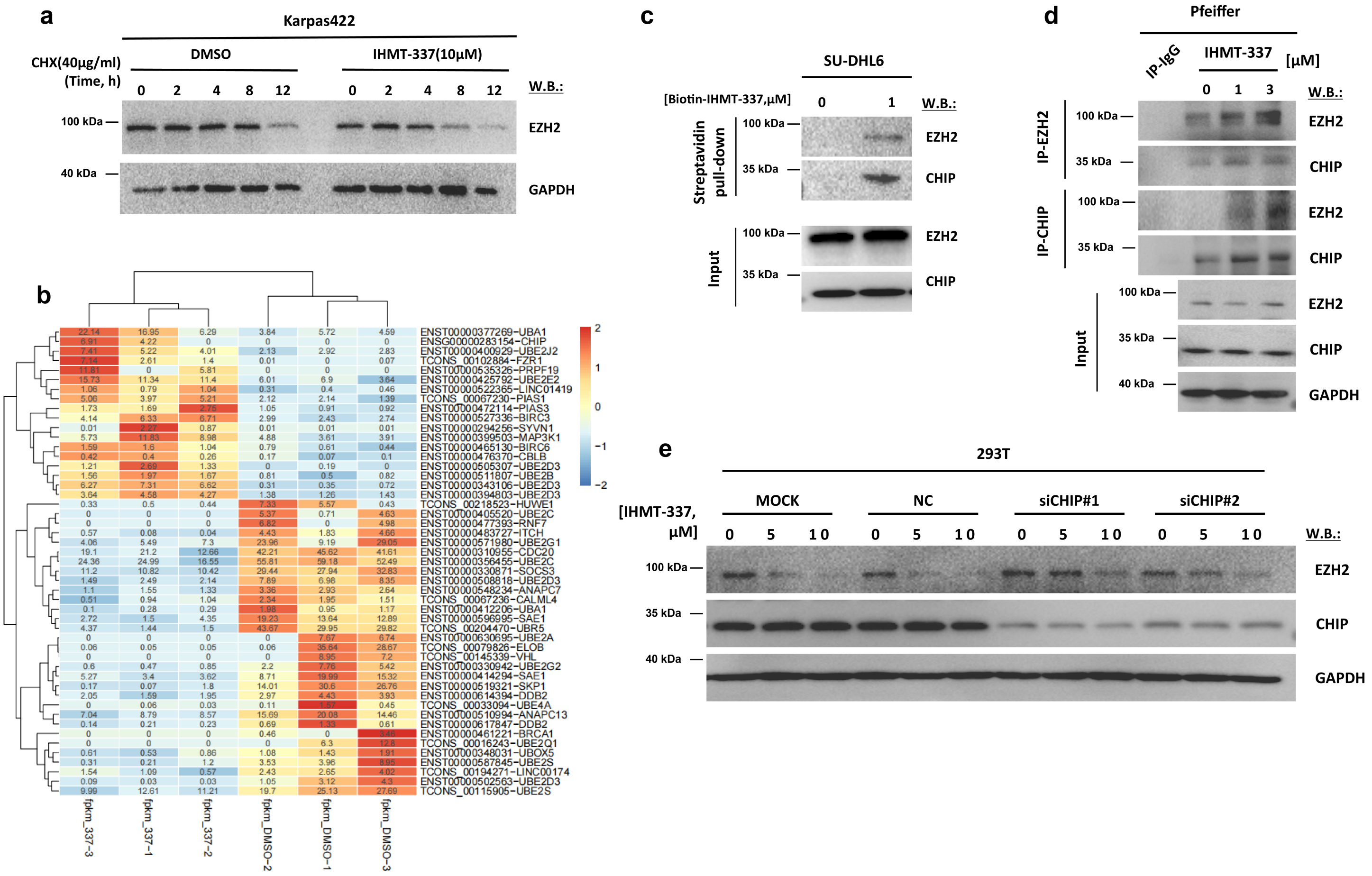

Supplementary Figure S3. Transcriptome analyzing identified CHIP as the E3 ligase that mediate EZH2 degradation post-IHMT-337 treatment.

a. Effects of 24-hr IHMT-337 treatment on EZH2 protein levels in Karpase422 cells. **b.** The transcriptome sequencing and gene enrichment analysis of Pfeiffer cells with or without IHMT-337 treatment at 10μM for 24h. **c.** Cell lysates from SU-DHL6 cells were treated with Biotin-IHMT-337 for 4hr at 0, 1μM. IP was performed with Streptavidin bead through streptavidin-biotin interaction, and immunoblotting was performed with antibodies against EZH2 and CHIP. **d.** co-IP assay: Cell lysates from Pfeiffer cells were treated with IHMT-337 for 24hr at 0, 1, 3 μM, EZH2 immunoprecipitation (IP) were performed with anti-EZH2 antibody and IgG beads, and immunoblotting was performed with antibodies against EZH2 and CHIP. **e.** Knockdown studies: Knockdown assay of CHIP in HEK-293T cells, then cells were treated with or without IHMT-337 (0, 5, 10 μM) for 24h, EZH2, CHIP and GAPDH protein levels were detected by western blotting.

Supplementary Figure 4

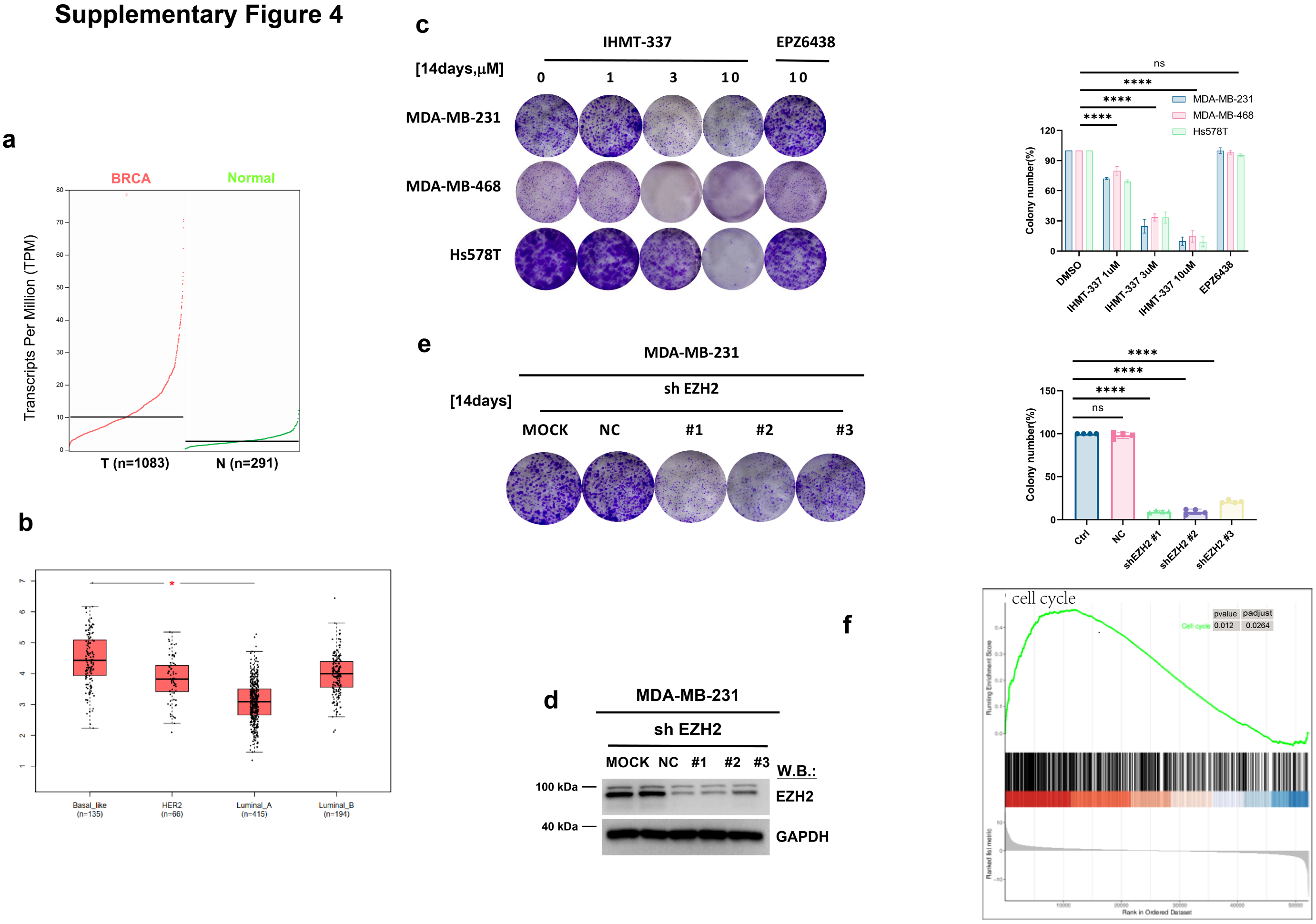

Supplementary Figure 4. EZH2 degradation caused by IHMT-337 influenced the proliferation of TNBC cells.

a and b. Expression level of EZH2 in different breast cancer types in the TCGA database. **c.** The clone formation was performed in TNBC cells following IHMT-337 treatment. **d.** The knockdown efficiency of EZH2 shRNAs in MDA-MB-231 were determined by westerns. **e.** The clone formation was performed in TNBC cells following EZH2 genetic deletion. **f.** Gene Set Enrichment Analysis (GSEA) of Pfeiffer cells post-treatment of IHMT-337.

Supplementary Figure 5

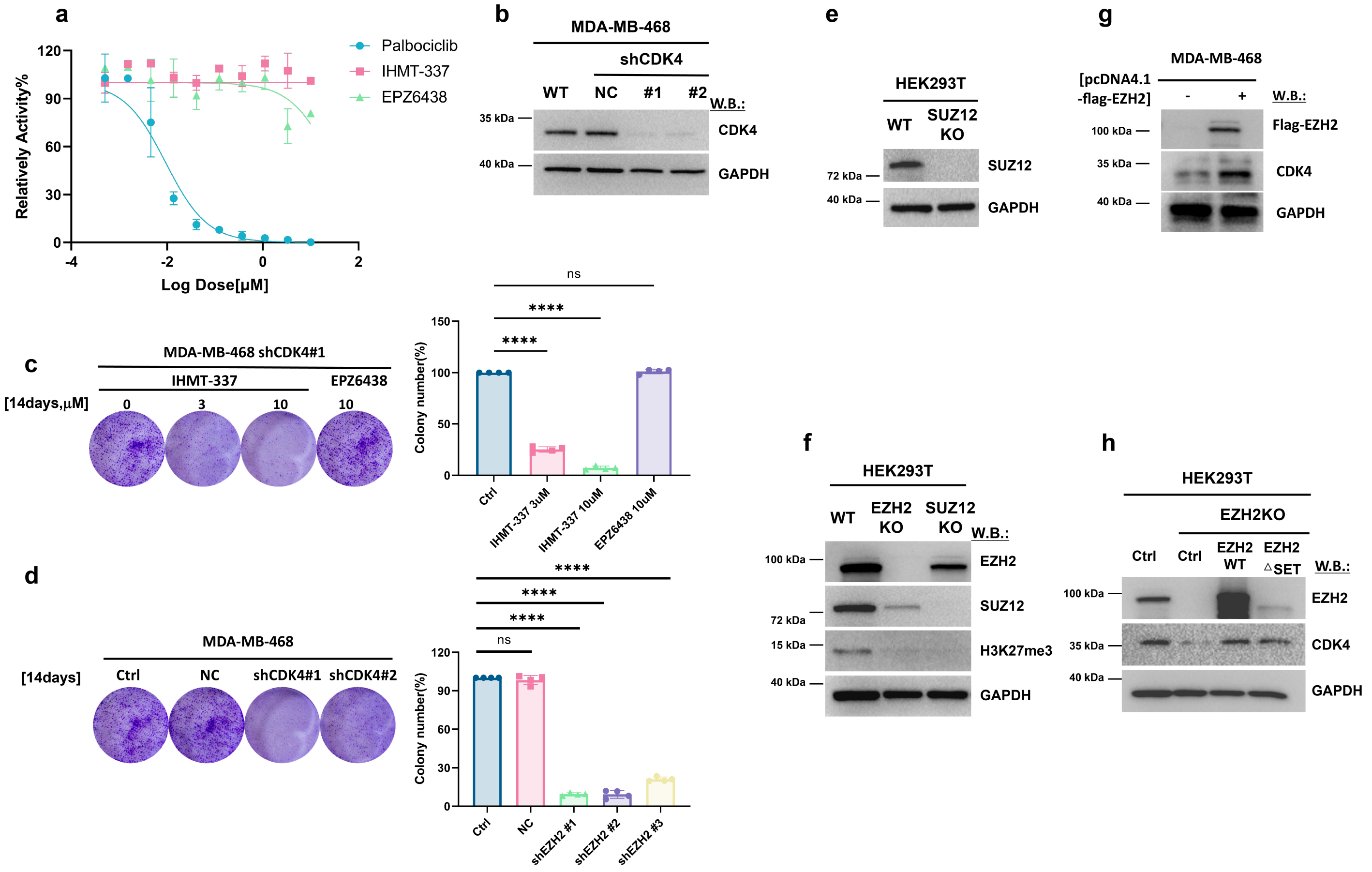

Supplementary Figure 5. EZH2 is a CDK4 transcription factor.

a. Biochemical assays of IHMT-337 against purified CDK4 kinase. Palbociclib was set as control. **b.** The knockdown efficiency of CDK4 in MDA-MB-468 were determined by westerns. **c.** The clone formation was performed in TNBC cells following CDK4 genetic deletion with or without IHMT-337 treatment. **d.** The clone formation was performed in TNBC cells following CDK4 genetic deletion. **e and f.** HEK293T cells were KO with sgRNAs of EZH2 and SUZ12 genes, the indicated proteins were determined by westerns. **g.** MDA-MB-468 cells were over-expressed with Flag tagged EZH2, the indicated proteins were determined by westerns. **h.** HEK293T EZH2 KO cells were over-expressed with EZH2 and EZH2-△SET, the indicated proteins were determined by westerns.
